# Distribution and Fate of Polyethylene Microplastics Released by a Portable Toilet Manufacturer into a Freshwater Wetland and Lake

**DOI:** 10.3390/w16010011

**Published:** 2023-12-20

**Authors:** Julie R. Peller, Gavin Tabor, Christina Davis, Chris Iceman, Ozioma Nwachukwu, Kyle Doudrick, Antigone Wilson, Alyssa Suprenant, David Dabertin, Jon-Paul McCool

**Affiliations:** 1 Department of Chemistry, Valparaiso University, 1710 Chapel Drive, Valparaiso, IN 46383, USA;; 2 Department of Civil and Environmental Engineering and Earth Sciences, University of Notre Dame, Notre Dame, IN 46556, USA;; 3 Dabertin Law Offices, 5246 Hohman Avenue Suite 302, Hammond, IN 46320, USA;; 4 Department of Geography and Meteorology, Valparaiso University, 1809 Chapel Drive, Valparaiso, IN 46383, USA;

**Keywords:** microplastics, sediment, plastic pollution, water pollution, air pollution, point source pollution, remediation

## Abstract

A portable toilet manufacturer in northwest Indiana (USA) released polyethylene microplastic (MP) pollution into a protected wetland for at least three years. To assess the loads, movement, and fate of the MPs in the wetland from this point source, water and sediment samples were collected in the fall and spring of 2021–2023. Additional samples, including sediment cores and atmospheric particulates, were collected during the summer of 2023 from select areas of the wetland. The MPs were isolated from the field samples using density separation, filtration, and chemical oxidation. Infrared and Raman spectroscopy analyses identified the MPs as polyethylene, which were quantified visually using a stereomicroscope. The numbers of MPs in 100 mL of the marsh water closest to the source ranged from several hundred to over 400,000, while the open water samples contained few microplastics. Marsh surface sediments were highly contaminated with MPs, up to 18,800 per 30.0 g dry mass (dm), compared to core samples in the lower depths (>15 cm) that contained only smaller MPs (<200 µm), numbering 0–480 per 30.0 g (dm). The wide variations in loads of MP contaminants indicate the influence of numerous factors, such as proximity to the point source pollution, weather conditions, natural matter, and pollution sinks, namely sediment deposition. As proof of concept, we demonstrated a novel remediation method using these real-world samples to effectively agglomerate and remove MPs from contaminated waters.

## 1. Introduction

Microplastics are ubiquitous pollutants that have been detected in all studied environments, from ocean depths [1,2] and freshwater algae [3], to remote mountain snow [4], animals [5], plants [6], and human cells [7]. Mostly described as pieces of plastic in the size range of 1 micrometer to 5 mm, microplastics’ origins are as extensive as the uses of plastic materials [8]. Common origins of microplastics include residential, industrial, and commercial wastewaters [9]. Washing and numerous other activities associated with textiles and other products create enormous amounts of synthetic and semi-synthetic microfibers that enter wastewater and ultimately contaminate surface waters and other environments [10,11]. Outdoor sources of microplastics include litter [12], road wear [13], and a myriad of other plastic debris and materials, which are released directly as microplastics or as larger forms of plastic that fragment into smaller pieces over time [14]. Numerous studies have established the threats and negative impacts of microplastics on living organisms [15,16]. Microplastics can bioaccumulate in organisms, and the effects are dependent upon the size and shape of the particles [17]. The uptake of microplastics by aquatic organisms can lead to blockages as well as harmful effects at the cellular and molecular level, but these vary according to the organism and feeding mechanism [18].

The types and quantities of microplastics in the environment often correlate with plastic sources, human activity, and transport mechanisms [19,20]. Outdoor plastic pollutants can wash into stormwater and/or scatter via the atmosphere, where they readily travel to other areas of the environment [21]. A number of studies have quantified microplastics in the atmosphere, one of many indicators of the ease with which lightweight pollutants disperse [22,23]. Polyethylene and polypropylene film fragments have been commonly found in sediments and soils, which is related to their widespread application in agriculture and landscaping to reduce weed growth [24,25]. Studies have shown farm fields that are fertilized with biosolids from wastewater treatment plants are land reservoirs of microplastics that can be further transported through agricultural field run-off [26]. A recent analysis proposed that the load of microplastics in agricultural soils could exceed six million tons [27]. Surface waters are well documented as a major means of transport and transformation of microplastic pollution and add to the microplastic load in the ocean [28].

While most plastic pollution originates from non-point sources [29], there are notable point sources, such as manufacturing processes and transportation/storage, that release plastics and microplastics in concentrated amounts. An example of an extreme point source of microplastic pollution was the X-Press Pearl maritime disaster in the Sri Lankan Sea in 2021. This disastrous explosion released an estimated 50 billion plastic pellets and other toxic chemicals [30,31]. More commonly, numerous plastic production facilities have been and continue to be point sources of polymer nurdles, or plastic resin pellets [32]. The contamination near a number of these facilities has been quantified, including the coastal bend region of southern Texas [33] and the west coast of Sweden [34].

The processing of plastic materials constitutes another point source of microplastic pollution. The contaminated area described in this study is a direct consequence of the manufacturing of polyethylene portable toilets and other restroom products by a company bordering George Lake in Hammond, Indiana (USA), PolyJohn Enterprises Corporation. Their manufacturing process creates and releases microplastics, which have moved into the adjacent freshwater marsh and lake for at least three years, according to documents of water quality violations by the Indiana Department of Environmental Management (IDEM) beginning in 2020 [35]. The state government cited the company for violations of the Clean Water Act, and it was directed to clean up the microplastic pollution and institute provisions to “insure containment of plastic materials” [36]. Since microplastics embed in sediment [37], adsorb to plants [3] and other living tissue [38], and readily move through aquatic environments [39], remediation of this pollution is a difficult endeavor. Efforts by the company and the city to remediate the pollution have included the placement of booms (also made of plastic and disintegrating over time), the suctioning/removal of contaminated water, and the placement of tarps along the property fence line. As of July 2023, this research team continued to observe large amounts of microplastics throughout the marsh, lake, and adjacent land after the company was again cited in June 2023 for “violation of the Agreed Order” [40].

George Lake is a shallow freshwater lake located on the Calumet Lacustrine Plain, approximately one mile inland of the modern shoreline along the southwestern edge of Lake Michigan. The eutrophic water body is divided into two basins, north and south, by a causeway with the George Lake Trail (formerly 125th Street). Today, the lake has a surface area of nearly 125 acres, an average water depth of 0.55 m (1.8 ft), and a watershed of 350 acres. The North Basin sediments consist of muck up to 0.15 m (0.5 ft) in depth overlying sands that extend to depths of 4.5–6.1 m (15–20 feet) [41]. George Lake has experienced major post-colonial modifications. More than half of the lake and the adjacent wetlands have been filled for residential and industrial development. The small size of the lake’s drainage area relative to its size means there is less opportunity for long-distance pollution transport to George Lake. The low topographic gradient of the surrounding landscape also reduces the possibility of long-distance contaminant transport due to low water flow velocities. Therefore, only close-proximity land sources can effectively discharge pollution into the lake and wetland.

Microplastics are not the only pollution in the George Lake ecosystem. The industrial history and field testing of George Lake sediment and its adjoining marsh detail numerous legacy contaminants, many of which remain in the ecosystem. Recent chemical analyses of collected sediment by this research team support the presence of numerous other chemical contaminants, including polycyclic aromatic hydrocarbons and other organics. (Details are provided in the Supplemental Information.) Adjacent to George Lake to the northeast is the property of the former company Federated Metals, which functioned as a metal smelting, refining, recovery, and recycling business beginning in 1937 [42]. In addition to chemical contaminants on the company property, it was reported that the company also dumped production slag into the lake, part of which acted as fill to expand the property area on the lake’s northeastern edge [41]. Even though Federated Metals closed in 1983, subsequent companies continued the industrial processes. This industrial legacy pollution site was recently proposed for the United States Environmental Protection Agency’s Superfund National Priorities list [43]. Most recently, the testing of at least 130 residential properties near the facility and areas of George Lake showed elevated lead levels [42]. The presence of these contaminants may influence the physical and chemical changes of the more recently emitted plastic pollution over time [44,45].

The aim of this study was to collect and evaluate water, sediment, and atmospheric samples for microplastic waste created and released by a company adjacent to the George Lake wetland in Hammond, Indiana, USA. Samples were collected near and away from the pollution source, at different depths in the sediment, and from the atmosphere. Data were analyzed to ascertain the loads, movement, and fate of the concentrated microplastic pollution. Additionally, experiments were performed to determine the potential for a novel remediation method for natural waters contaminated with microplastics.

## 2. Materials and Methods

### 2.1. Study Sites

**Figure 1.**
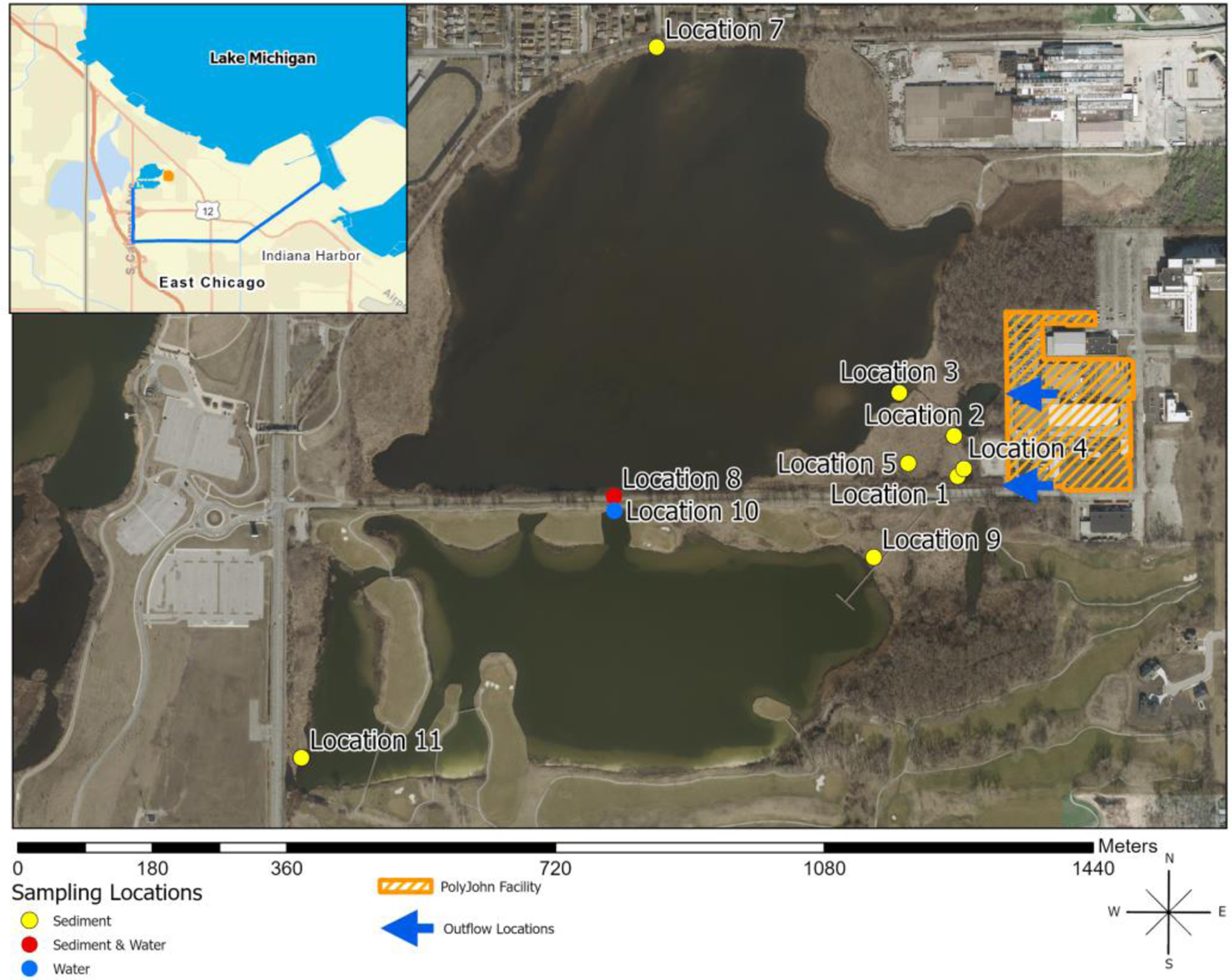
Map of the area of contamination, manufacturing facility, and sampling locations. The upper-corner map shows George Lake and its tributary to Lake Michigan. The orange mark near George Lake is the PolyJohn company. Note that the outlet canal water sample (Location 12) is not shown on the map but is approximately 1100 m south of Location 11. (Locations 1 and 2 = outflows; Location 3 = George Lake adjacent to the marsh; Locations 4, 5, 6 = marsh; Locations 7–11 = George Lake away from the marsh).

Water and sediment samples were collected from George Lake, which is in the far northwest corner of the state of Indiana (USA), and its adjoining marshes and outfalls. The small lake, composed of two basins, is situated between Wolf Lake and Lake Michigan (41°40′16″ N 87°30′5″ W), approximately 20 miles southeast of Chicago, Illinois. George Lake water flows south to the George Lake Canal and eventually northeast to Lake Michigan, one of the Laurentian Great Lakes (see Figure 1 inset). The lake has a very narrow transition to upland or developed areas on the northern and southern margins, typically less than 10 m in width. On the eastern and western edges are marshes that extend 100–150 m from the water edge to upland areas. Surface water input to the lake from the watershed is via subterranean waterways that drain the surrounding developed landscape. Three outflow locations are on the north and northeast sides of the north basin, which drain the residential and former metals industrial areas.

Two drainage outfall locations are in the southeastern corner of the lake, which largely drain the PolyJohn facility and adjacent college. Water and sediment were collected in the spring (April/May) and autumn (October) from 2021 through spring 2023 and were recorded as Outfalls 1 and 2 on Figure 1 (Locations 1 and 2), George Lake (Location 3), and Marshes 1 and 2 (Locations 4 and 5). The marsh samples were mostly aqueous and processed as water samples. Core sediment samples were taken in June and July 2023 at depths up to 65 cm to assess the movement of microplastics vertically in the sediment. Lake water samples in spring and autumn 2021–2023 were collected from the basin of the lake where the marsh empties into the lake (Figure 1, Location 3). During the summer of 2023, additional lake samples were collected on the southern shore of the lake at the culvert that flows into the south basin (Location 8), from the northern edge of the south basin (Location 10), and from the outlet canal that drains the south basin to the southwest and ultimately joins the George Lake Canal (Location 12—not shown on the map in Figure 1). Sampling locations are shown on the map in Figure 1.

### 2.2. Sample Collections

Water samples were collected into pre-rinsed 500 mL glass bottles, obtained either directly from the water or with an extension pole. Surface sediment samples represented the upper 5 cm of sediment at the vegetated shorelines and were collected directly into 100, 250, or 500 mL pre-washed and rinsed glass bottles.

Initial sediment deposits were investigated within the marsh at the southeastern corner of the north basin using a 2″ auger to determine appropriate sampling approaches. Samples were then collected using an AMS sludge and sediment sampler with stainless steel sampling tubes and a slide hammer. All samples were stored in a cooler or cooled vehicle and transferred to a refrigerator within an hour until they were processed in the laboratory. Since most of the samples had readily visible polyethylene microplastic contamination, blank samples were only taken in the field where MPs were not highly visible.

Airborne particulates were sampled from the air mass directly to the west of the facility at ground level with a particulate matter monitor via active and passive aerosol collection techniques. Active aerosol collection was undertaken through mechanical pumping using a 3 L/min vacuum pump remotely operated with battery power. The sampler was located approximately 4 m from the facility fence to capture any lofted particulates from the facility exhaust. The active sampler operated for approximately two 24 h sample runs, filtering the air mass with a filter holder from Millipore Swinnex 25 (MilliporeSigma, Burlington, MA, USA) and through a 0.8 µm cellulose filter from Tisch Scientific, SF15108 (Cleves, OH, USA) to capture particulates above 1 µm in size and to approximately 10 µm. After active sampling runs, the recessed active sampler lid operated as a passive sampler over seven days to collect particulates too large in size to be captured by the active sampler.

### 2.3. Laboratory Processing of Field Samples

Laboratory blanks were performed alongside the lesser contaminated surface water samples and deep sediment samples. External contamination was deemed negligible for most of the field samples that contained a heavy load of microplastics. Polyethylene microplastics were not detected in the blank samples. Synthetic microfibers, which are common microplastic contaminants, were not counted as microplastics for this study but were observed in most samples.

Core sediment samples were processed and analyzed for physical parameters in the Physical Geography Laboratory at Valparaiso University. Cores were extruded from the sampling tubes using wooden dowels into galvanized trays and sub-sampled in 5 or 10 cm increments, based on visual interpretation of organic and mineral sediments, and placed into glass beakers. (See Supplemental Information). Samples were dried overnight at 100 °C and disaggregated using ceramic mortar and pestle. Sub-samples for each depth were removed using stainless steel spatulas and submitted for processing to isolate microplastics. The remaining sample volumes were sieved using stainless steel 2.0 mm mesh sieves to determine gravel percentage. The under 2 mm fraction was sub-sampled for loss-on-ignition and particle size analyses. Loss-on-ignition was performed in ceramic crucibles on 10 g subsamples, dried overnight at 100 °C and organic content determined by heating to 500 °C for 3 h in an oven (Fisher Scientific, Waltham, MA, USA, Isotemp Muffle Furnace 550 Series Model 126). Samples with organic contents greater than 5% had organic matter removed using sodium hypochlorite following NRCS method 3.2.1.2.1.1.1.2 [46], using deionized water and centrifugation at 4500 rpm for 5 min. Sediment subsamples for particle size analysis were allowed to sit overnight in a 50 g/mL solution of sodium hexametaphosphate (HMP) to deflocculate aggregates.

Water and sediment samples were processed to isolate and analyze microplastics in the Environmental Chemistry Laboratory at Valparaiso University. All laboratory glassware was cleaned and rinsed several times with deionized water. The volume of lake/marsh water filtered ranged from 10 mL to 500 mL, depending on the load of solids. Sediment samples were transferred directly to glass beakers, or the glass collection bottles were lightly covered with foil and set in an oven at ~90 °C for 1–3 days until the contents were dry. The sediment was pulverized and sieved if it contained large, natural pieces. Samples of 30.0 g dried mass (dm) (or less when the amount of sample was limited) were transferred into 250 mL beakers and covered with foil. A volume of 100–300 mL of water was added to each sediment sample for density separation since polyethylene is less dense than water and the microplastics floated on the water surface. The mixture was thoroughly stirred by hand and then for 30 min on a stir plate with a magnetic stir bar. The mixture was left overnight to settle out the heavier particles. The liquid portion of the aqueous mixture was decanted through a vacuum filter funnel containing a 5.0 µm pore size, 47 mm nylon filter disc. The filter paper and contents were placed into a 40 mL glass centrifuge tube.

Nylon filters containing the microplastics and other solids were oxidized to reduce natural organic material. A small magnetic stir bar was added to a 40 mL centrifuge tube along with the collected solids. A 3:2 solution of DI water and hydrogen peroxide (Sigma-Aldrich Chemical Company, St. Louis, MO, USA ACS reagent, 30 wt. % with inhibitor) was added to the centrifuge tube. Control blanks of only filter paper were used when deep sediment samples and open water samples were processed. The mixtures were heated at 70 °C, vigorously stirred using a stirrer/hot plate, and exposed to a UV medium-pressure lamp (Ace Glass, Vineland, NJ, USA, Power Supply 7830–60, UV power 450 W, ) for 60 min. Hydrogen peroxide, in the presence of ultraviolet light, forms hydroxyl radicals, an effective reactant for the oxidative breakdown of natural organic materials [47]. After 60 min, the samples continued to stir at room temperature until the oxidative reaction was visibly complete. The oxidized aqueous mixture was filtered through a clean 5.0 μm pore size, 47 mm nylon filter disc, then transferred to a glass Petri dish and covered. For a few of the samples containing heavy loads of solids, the oxidation process was repeated.

The collected air particles were subjected to the same oxidative cleanup as the soil and water samples.

### 2.4. Quantification and Characterization of Microplastics and Soil Particles

Isolated microplastics were viewed under various stereomicroscope magnifications (7–40×), which limited the detection of microplastics to approximately 20 µm and larger. For the water and sediment samples analyzed through March 2023, smaller portions of the processed microplastics were manually counted and extrapolated to the larger sample size. Fiji software (Version 2.9.0) was implemented for the quantitative counting of microplastics from samples collected in 2023 [48].

The chemical identity of many microplastics collected in the field was verified using Raman and infrared spectroscopy. Both cleaned and unprocessed microplastics were analyzed with a Renishaw inVia Qontor confocal Raman microscope (Renishaw Inc., West Dundee, IL, USA) and compared to medium density polyethylene (MilliporeSigma, Burlington, MA, USA, with an average molecular weight (MW) ~4000 g mol^–1^, an average Mn ~1700, as determined by gel permeation chromatography, and a density of 0.92 g/mL at 25 °C). The inVia Qontor was equipped with a Leica DM2700 optical microscope (Leica Microsystems, Wetzlar, Germany) using brightfield microscopy. Images of microplastics were collected with a 20× or 100× objective, and Raman spectra were collected with a 20× long working distance objective using 532 nm excitation. The exposure time was typically 3 s, and the number of accumulations varied between 5 and 20 for adequate signal-to-noise. Spectra were baseline subtracted to remove noise and an uneven background. Bulk samples of the rotopowder collected in the field were analyzed using a Nicolet iS50 FTIR (Thermo Scientific, Waltham, MA, USA) where 16 or more scans were collected with a resolution of 4 cm^−1^ and compared to the medium density polyethylene standard.

The sizes of the soil particles were measured using a Horiba LA960 laser diffraction particle size analyzer (Horiba, Kyoto, Japan).

### 2.5. Lab-Scale Remediation of Microplastics in Natural Water

Lab-scale experiments were performed first using 500 mL flasks, 200–300 mL of water, and a liquid compound to agglomerate the microplastics. A magnetic stir bar was added to the mixture, and the flask was set on a stir plate at a high rate of rotation. For larger volumes of water (600 mL), a jar tester (Phipps & Bird 7790–400 Six-Paddle Stirrer, Richmond, VA, USA) was used to stir the mixtures in 1000 mL beakers. In both cases, the water medium was mostly the natural water from George Lake or the marsh. To increase the water volume, tap water was added. The specifics of these experiments are currently under review as a patent application [49].

### 2.6. Data Analysis and Maps

Results were shared among project participants using Google Sheets, with quantification carried out using sheet calculations. Spatial analysis, mapping, and some figures were performed using ArcGIS Pro 3.1.3. Other figures were made with Python 3.7.7, principally using the numpy, pandas, and matplotlib libraries.

## 3. Results

### 3.1. Point Source Polyethylene Microplastics Pollution Detection and Material Verification

**Figure 2.**
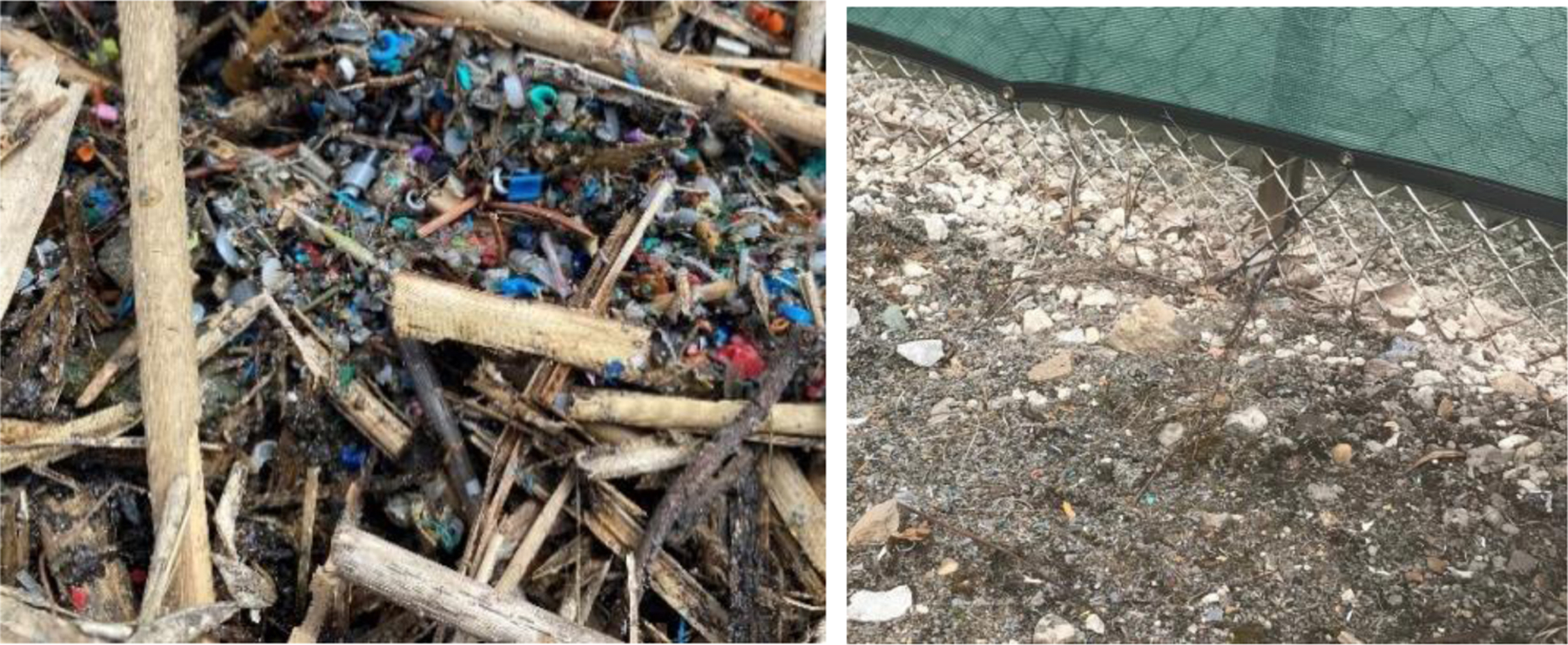
Photographs of microplastics scattered in the marsh sediment of George Lake in Hammond, Indiana (USA) (**left**) and at the property line (**right**). The right photo shows the fence at the edge of the company’s property where plastic particles, particularly fine, gray microplastics, moved into the wetland property.

Point source microplastic pollution along and near the fence of the PolyJohn Enterprises Corporation property (Figure 1) in Hammond, Indiana, USA, where polyethylene and polyethylene powder are processed to manufacture portable toilets and other items, was studied from 2021 to 2023 [50]. The microplastics contamination was not contained by the manufacturer and moved into the adjacent wetland and lake, as shown in Figure 2 photographs. The pollution has been documented by this research team, local citizens, local media [51], and the Indiana Department of Environmental Management (IDEM) [52]. The highest amounts of microplastics have been observed outside the company’s fence that separates it from the wetland. The photographs in Figure 2 were taken at the property fence and in the marsh in the spring of 2022, confirming the source and extreme amount of microplastics released into the natural area by the PolyJohn company. Throughout the three years of sampling in and around the wetland and George Lake, microplastic contamination was readily visible, even after numerous citations and fines by IDEM and after remediation measures by the company and city [51].

**Figure 3.**
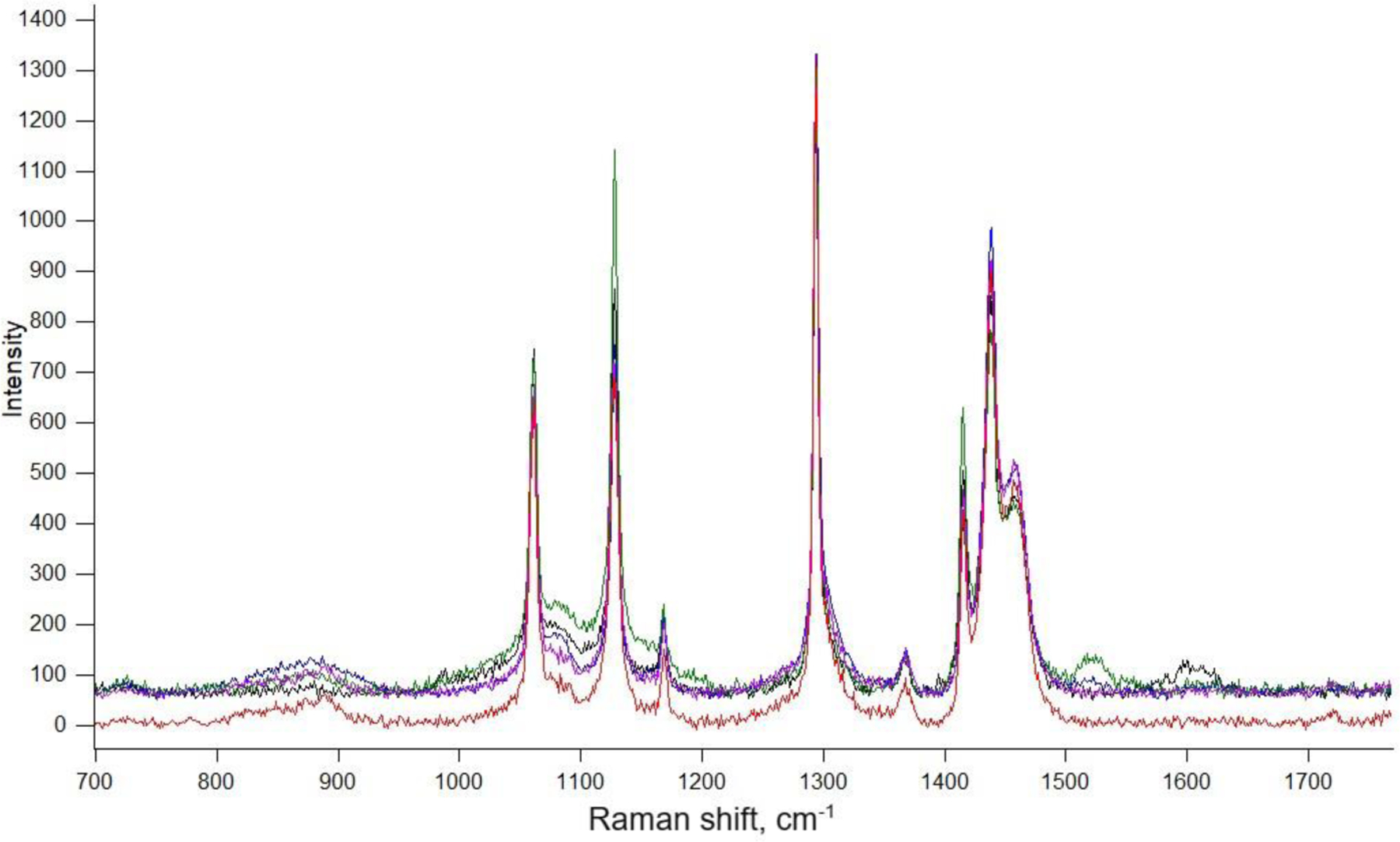
Raman spectra of the polyethylene standard (red trace), colored particles (black and green lines), and rotopowder particles collected from George Lake (blue line).

Numerous pieces of microplastics collected from the contaminated area were verified as polyethylene using Raman and IR spectroscopy (IR spectra can be found in the Supplemental Information). The colored particles were mostly curved shavings that ranged in size from 500 µm to 5 mm; however, nurdles were routinely observed and collected as part of the field samples. Figure 3 shows the Raman spectra for three different microplastics collected from the field and the polyethylene laboratory standard. For the field samples, minor variations in the spectral signatures, likely due to absorbed/adsorbed substances, additives, or a specific type of polyethylene, were noted when compared to the laboratory standard sample of medium-density polyethylene. Smaller particles of PE, often termed rotopowder, used for molding in manufacturing, were also isolated and characterized as polyethylene. There were minimal or no indications of chemical oxidative weathering, mostly defined as the presence of C=O bands [53], from the spectral analysis of over 20 microplastic pieces. In Figure 3, the spectra of the MPs exhibit no C=O bands, which appear around 1700 cm^−1^.

### 3.2. Quantification of Microplastics in Water, Sediment, and Air Samples

**Figure 4.**
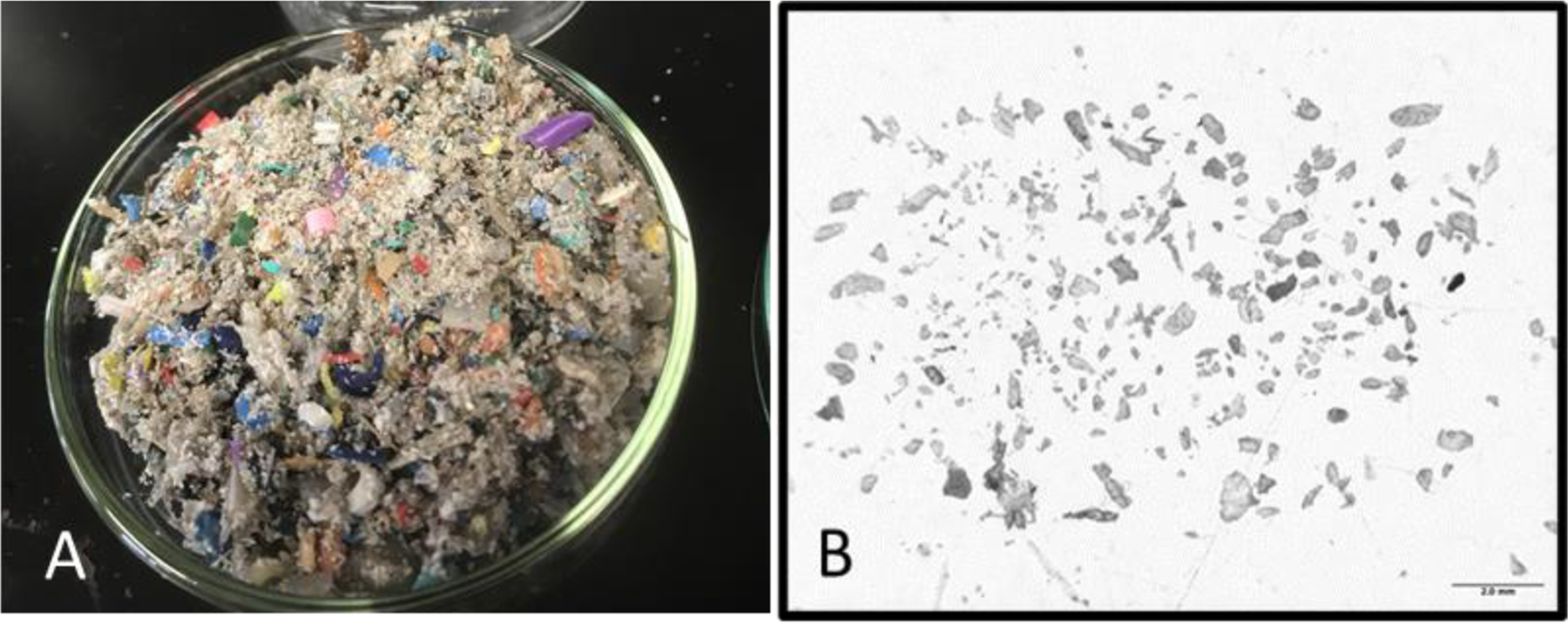
(**A**) Microplastics recovered from a 100 mL marsh water sample in April 2022, consisting of both large and small polyethylene microplastics. (**B**) Magnified view (7×) of isolated rotopowder microplastics. The scale bar is 2.0 mm.

The initial water and sediment samples collected in the spring of 2021 contained large amounts of colored microplastic particles of varying shapes and sizes. The average number of microplastics recovered in the 2021 samples was approximately 700 MPs per 100 mL of marsh water and 1000 MPs per 30 g (dm) of sediment. Smaller and colorless/gray particles isolated from these samples were analyzed using infrared spectroscopy and were mostly identified as silica sand particles. One year later, in April 2022, the field samples contained far more microplastics and a larger number of smaller particles. Different from the previous year, the smaller particles collected in 2022 were identified as polyethylene, or rotopowder. These were isolated and observed in all samples, often in large quantities, in 2022 and thereafter. Figure 4A shows the intense amount of microplastics isolated from a 100 mL sample collected from the marsh water in 2022. Over 100,000 microplastics were quantified by analyzing a portion of the samples (usually 10%) and extrapolating to the entire sample. Figure 4B is a magnified image of the isolated rotopowder. These smaller gray microplastics, different from the colored microplastics, typically ranged in size from 20 µm to 500 µm.

**Figure 5.**
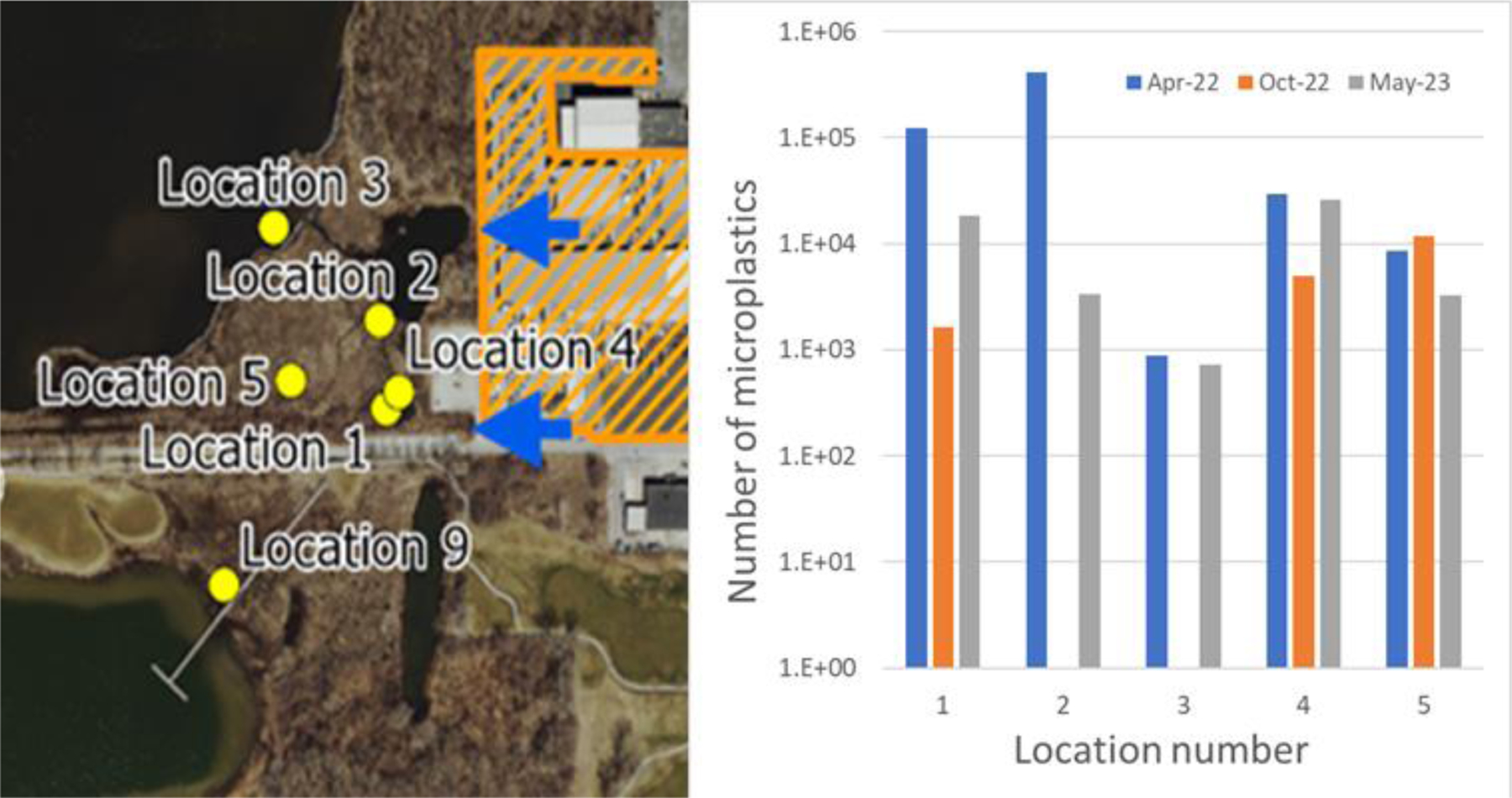
Counts of microplastics in the water samples from samples collected from locations 1–5 in 2022 and May 2023, where locations 1, 2, and 4 were near the manufacturer. The heavy blue arrows on the map represent the flow of microplastics from the source. The number scale is a log 10 scale.

For the April 2022 water samples, the number of microplastics in 100 mL ranged from 570 from a lake water sample to 411,400, which were recovered from location 2, close to the source. Of the 123,680 microplastics isolated from another marsh water sample (location 1), 117,800 (95%) were the smaller particles (rotopowder). The number of microplastics in marsh sediment samples collected in April 2022 ranged from 16,280 to 42,380 per 30 g (dm). Figure 5 summarizes the data from the water samples (locations 1–5) collected in April 2022, October 2022, and May 2023. The much higher numbers of microplastics in marsh water samples compared to the George Lake samples (location 3) can be attributed to the closer distance to the source of the microplastics and the greater content of natural matter in the water that inhibits the dispersal of the microplastics.

Compared to the samples collected in April 2022 and May 2023, the samples collected from the drainage outfalls and the marsh sites in October 2022 had lower microplastic loads. Notably, marsh water samples from April 2022, one of which contained 411,440 microplastics/100 mL, had significantly higher amounts than other samples taken at these sites over the three-year study. There are likely several factors contributing to the extremely high amounts of microplastics in the water and sediment in April 2022. In the months prior to this sampling, it is conceivable that greater amounts of point source plastic debris moved into the wetland. Weather conditions in the spring are additional factors that can influence MP transport and local loads in the water and sediment [54]. Microplastics released by the company during the winter months likely accumulated when the ground was frozen and/or snow-covered, reducing their distribution. Typical spring season rainfall and wind might have transported the accumulated microplastics through the land and outfalls and into the marsh.

**Figure 6.**
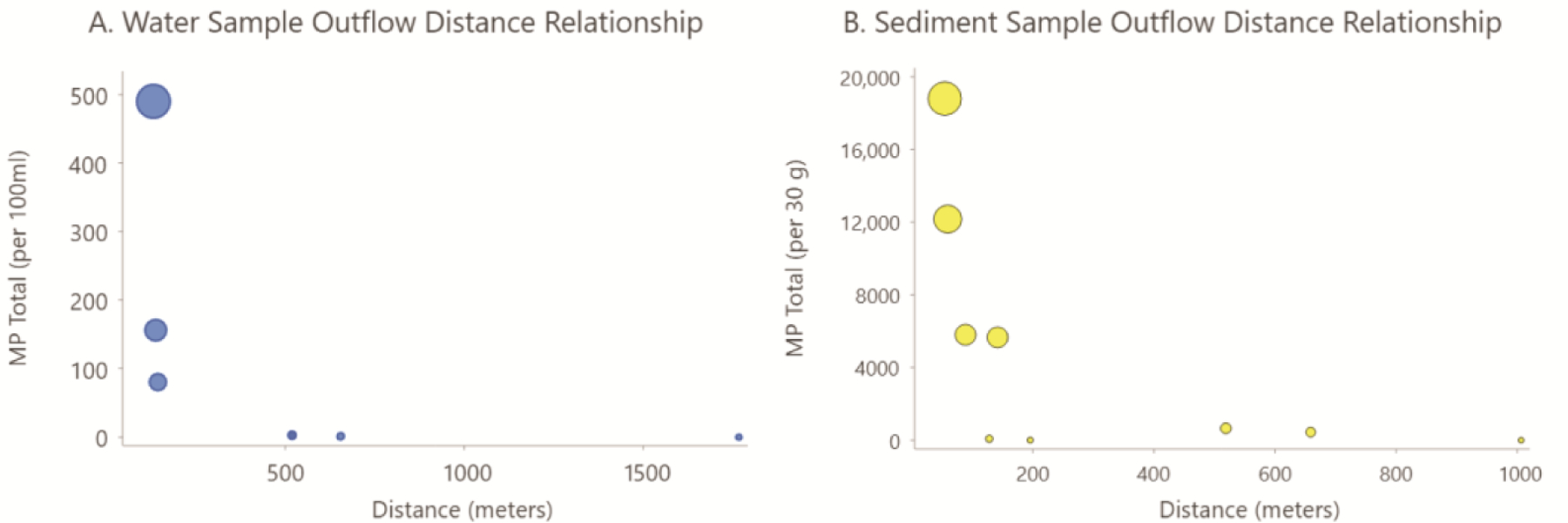
(**A**) Numbers of microplastics in George Lake water (blue circles) as a function of distance from the nearest outflow location. (**B**) Total number of microplastics per 30 g of dried sediment (yellow circles) as a function of distance to the nearest outflow point. For both graphs, the larger circles represent higher loads of microplastics. Different coloration between dots to emphasize different sample types, blue for water samples, yellow for sediment samples.

During the summer of 2023, additional water samples were collected in duplicates around the lake and farther from the source of the pollution. These sites are noted on the map in Figure 1 as Locations 8, 10, and 12. The water samples collected from the far shores (Locations 10 and 12), approximately 760 and 2070 m from the marsh discharge area, respectively, were found to contain either no microplastics or 1–7/100 mL. The field and lab blanks were clean. Figure 6 shows the average number of MP for the locations where water was collected at 10 m intervals away from the edge of the contaminated marsh (near Location 3) combined with the other lake water samples from Locations 7, 8, 10, and 12. From this small sample set, it is apparent that the number of microplastics recovered from the water dropped significantly as the distance from the source increased, especially after entering the lake. The data suggest that the low-density polyethylene microplastics readily disperse once they enter the open water. Water provides the means for further distribution of the microplastics until they encounter a temporary or permanent medium. Other studies of inland lakes similarly cite the many sinks, both temporary and long-term, of microplastics [55].

### 3.3. Sediment Depth Sampling and Air Monitoring

Since relatively low numbers of microplastics were isolated in the lake water samples, sediment was suspected to be an effective sink for the pollutants, and this has been well established in numerous studies [56]. Sediment from the shorelines of George Lake and sediment core samples taken in the marsh were collected and processed in the summer of 2023 to better understand the extent of distribution and fate of the microplastics. Core samples were collected at the marsh sites labeled Locations 1–5 on Figure 1. Sediment samples were taken at similar locations to the water collections around the lake and at locations farther from the pollution source (Locations 7–9, 11). The number of microplastics recovered from shoreline sediment across the lake from the PolyJohn company (Location 7) was 448 MPs/30.0 g (dm). Sediment from Location 8, which represents the sediment around the culvert passage to the south basin of the lake, contained 823 MPs/30.0 g (dm). The number of microplastics isolated in the sediment collected from the farthest two locations (9 and 11) from the marsh along the south basin shore, 760 and 950 m, respectively, varied from 0 to 5 MPs/30.0 g (dm). From this set of data, the number of microplastics deposited in the sediment of the contaminated north basin was significantly higher than the contamination in the south basin, suggesting the sediment contamination has been mostly limited to the lake receiving direct input. The culvert and its vegetation are at least currently acting as a constriction point that is inhibiting the transport of microplastic pollution from the north to the south basin.

**Figure 7.**
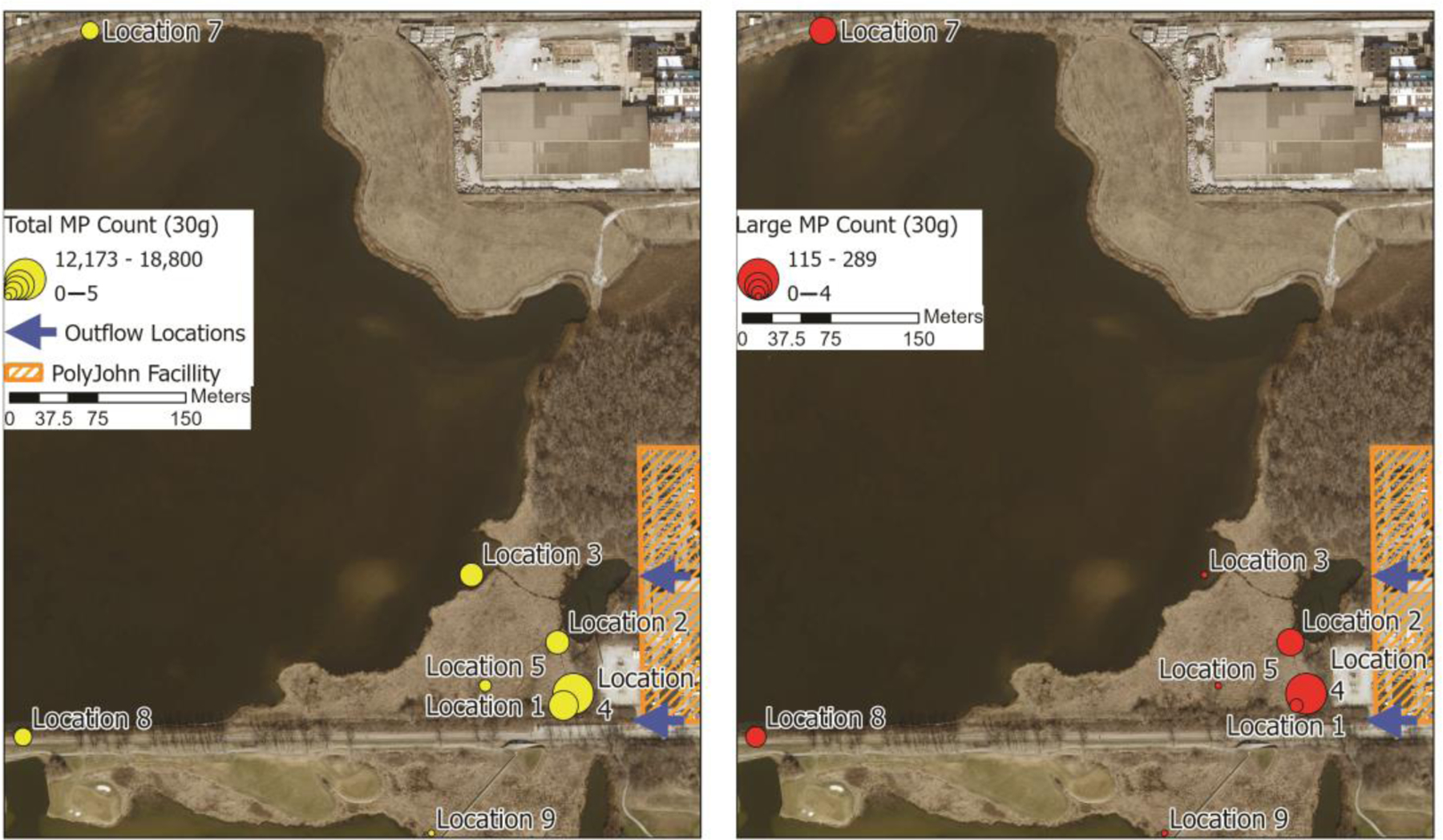
Microplastics count in sediment. The left map shows the total number of microplastics (rotopowder and larger microplastics combined) using graduated symbols. The numbers decrease with increasing distance from the outflow locations. The right map shows only the data for the larger microplastics, where larger counts are concentrated along drainage ways in the marsh or the lake waterline.

Microplastic pollution in sediment in the north basin is in close proximity to the source and also in direct flow paths of the water, according to our data. Total microplastic counts in the sediment decrease with increasing distance from the drainage outflow locations, as seen in Figures 6B and 7. It is notable that microplastics were still found in open-water lake sediment approximately 20 m from the shoreline, but that these plastics were exclusively rotopowder. Within the marsh, total numbers of microplastics showed a weak relationship to drainage ways, whereas larger numbers of microplastics showed an almost exclusive association to drainage ways and lake edges. This suggests that the much smaller rotopowder microplastics are more easily transported deeper into vegetated habitats during highwater stands and can become entrapped within depositing sediments more readily than larger particles.

**Figure 8.**
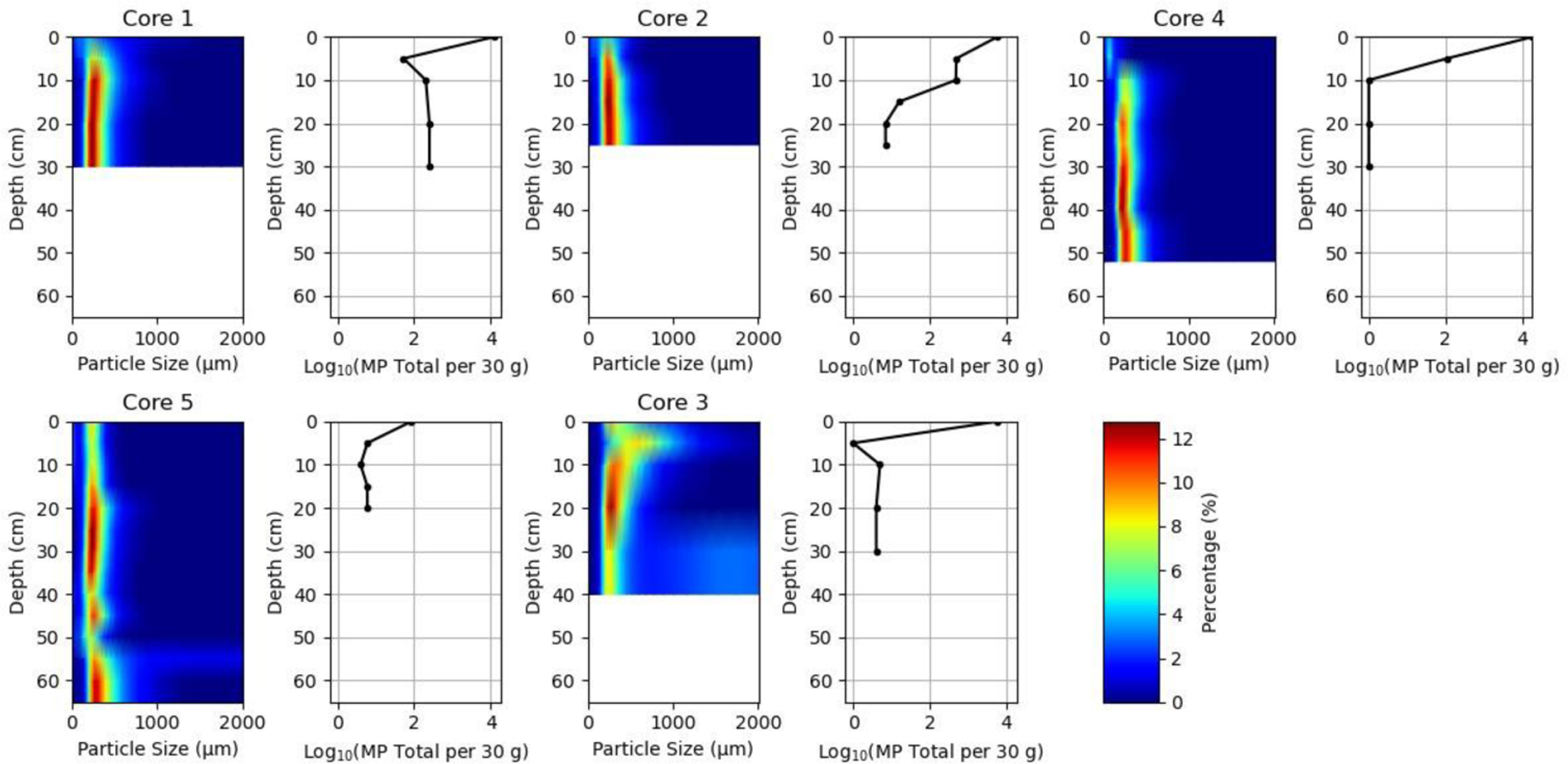
Core sediment particle size depth distributions as heatmaps alongside total microplastic counts normalized to 30 g of dried sediment.

The study of microplastics distribution across sediment depth shows that plastic pollution is largely, but not exclusively, concentrated near the surface. Larger microplastic fragment counts ranged from 0 up to 98 pieces per 30 g (dm), while rotopowder counts were in excess of 7000 for the same mass. As seen in Figure 8, microplastics were found to be concentrated in the top 5–10 cm of the surface. Rotopowder was found in subsurface samples, especially Locations 1 and 2, with counts up to 250 at depths of 30 cm. This is in contrast to similar sediments in the marsh (Locations 4 and 5) or in the lake (Location 3), where the microplastic counts were below 10 per 30 g. Locations 1 and 2 are very close to the source and are in contact with a greater amount of microplastics than locations away from the source. The data indicate that while most of the microplastics are moving horizontally into the marsh, some are migrating vertically.

Variations were noted in the number of isolated microplastics from the cores, despite similar surface organic contents and fine to very fine mineral sediment. For location 2, there was a high amount of rotopowder associated with high organic content in fine to very fine sands. Microplastics were able to move more readily into the subsurface in comparison to Location 3, with its coarser sediment, larger pores, and gravelly coarse-to-medium sands, despite similar counts in the surface sediment. This suggests that the lower densities and higher porosities typically associated with organic and organic-rich sediments preferentially support downward transportation of the smaller microplastics. This may make remediation of wetland areas polluted with microplastics more challenging than mineral sedimentary environments, especially if cleanup efforts are long after initial contamination.

Preliminary studies of microplastic contaminants in the air were carried out using both passive and active air monitoring set approximately 4 m from the PolyJohn fence line. The active particulate matter monitor collected particles above 0.8 µm in diameter over approximately 24 h on two different dates. The analyzed filters were inconclusive for particle identity. However, a wide variety of airborne particles of different sizes, shapes, and materials were collected from the passive sampling of the surface of the monitor lid over 7 days. These particles were analyzed using stereomicroscopy, Raman microscopy, and spectroscopy. (Data can be found in the Supplemental Information). Several particles in the size range of 10–30 µm matched plastic polymers, mostly polyethylene terephthalate in the Raman library, and not the PE plastic isolated from the ground-level samples. Polyethylene particulates created during the product manufacturing process (cutting and trimming) may not have a size domain that is loftable as an airborne particulate. Other larger particulates, approximately 500 µm in size, that were collected via passive sampling matched the Raman library for glassy carbon. It is possible that these carbon materials could be a result of thermoplastic resin breakdown after exceeding the thermal degradation limits [57].

### 3.4. Potential Remediation Methodology for MP Contamination in Surface Waters

The remediation measures by the PolyJohn company, which have included tarps on the fence and booms in the water, were put in place to prevent the further dispersal of the microplastics manufacturing waste from their property. From both visual inspection and sample analysis, these provisions have not stopped the movement of the microplastics from the company property into the natural wetland. The ineffectiveness of booms for the collection of larger plastic materials in rivers was described in a recent study [58]. Further, the use of booms requires follow-up measures to remove the particle pollutants in open waters, and they capture only floating debris [59]. Booms are less inclined to collect microplastics in wetlands that contain greater amounts of other solid matter. An additional remediation measure used by the local municipality involved a large vacuum hose to suction out the microplastics. However, this type of method removes the natural suspended components of the water (phytoplankton, macroinvertebrate larvae, and microorganisms) in addition to the contaminants, effectively stressing the ecosystem [60]. Remediating MP contaminants using nontoxic methods and without major disturbances to the natural water constituents is a major challenge that should begin with a feasibility study [60]. A number of newer technologies that mostly involve filtration have been outlined in a review by Fiore et al. [61]. For point source microplastic pollution, the most effective solutions involve upstream actions to eliminate the release into the environment, which often requires binding policies and enforcement [62].

**Figure 9.**
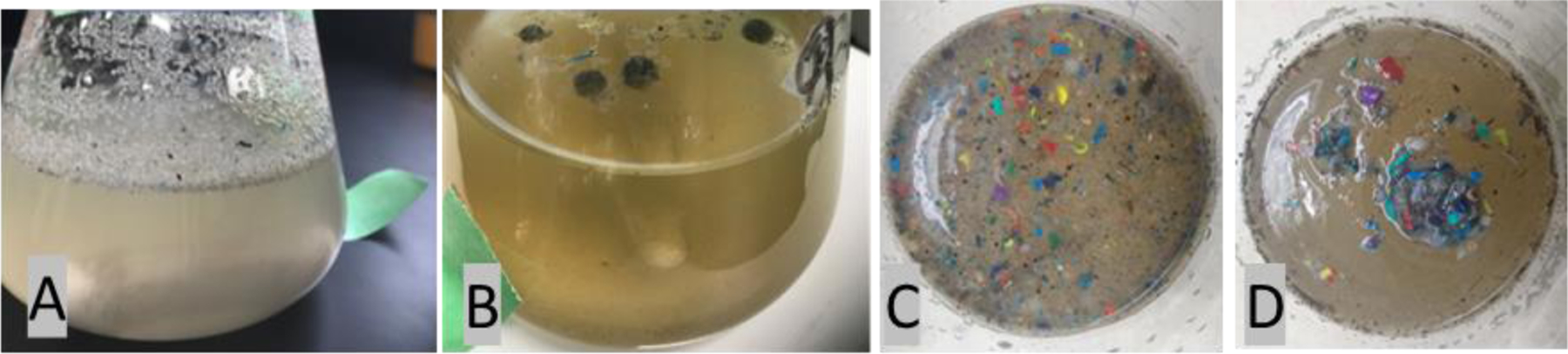
(**A**–**D**) Diffuse microplastics in water (**A**,**C**) and their agglomeration in water using a nontoxic, liquid hydrocarbon and an effective stirring mechanism (**B**,**D**).

As part of this study, water samples from the highly contaminated marsh were used in experiments to assess the success of creating larger formations of only microplastics that can be readily removed from the water. Figure 9A,B shows the mixture of mostly smaller microplastics in the marsh or lake water in a flask (200–300 mL) before and after the addition of a liquid hydrocarbon (~0.8%) and stirring with a magnetic stirring bar for approximately 12 h. A more diverse mixture of microplastics was tested on a larger scale, 600 mL, using a jar tester for 48 h, and the results are shown in Figure 9C,D. When the spheres of microplastics were removed, dissected, and analyzed using IR spectroscopy, over 95% of the material was identified as plastic. The natural particulates were left behind in the water.

Our experiments using a nontoxic, novel agglomerant suggest an effective way to collect microplastics from water without causing damage to the ecosystem. Currently, there are limited remediation measures to clean waters polluted with microplastics [63]. Challenges include the variation in sizes, shapes, densities, adsorbed films, water properties, and the numerous other interactions of microplastics. Further, once micro- and nanoplastics are released into the environment, their distribution is minimally predictable. The high loads of microplastics isolated in this study from the marsh sediment and the north lake sediment indicate that the particle pollutants move out of the water and settle in the sediment, either temporarily or permanently. Our remediation methodology shows promise to be effective in removing microplastics from water. For this point source pollution, the marsh water would be the target of remediation since it contained high loads of microplastic waste. This would involve temporarily removing and processing marsh water to remove the vast majority of the microplastics, followed by the return of the water and its natural contents to the marsh.

### 3.5. Study Limitations

A number of factors influence the distribution and fate of microplastics in a wetland and small lake environment. This study did not evaluate the role of the abundant plant life in the wetland, which changes seasonally. Numerous studies demonstrate the effective interactions of MPs with plants and plankton, and researchers have even proposed the use of plants for the remediation of these particulate pollutants or as a bioindicator of their presence [64,65]. Also missing from the study were samples from the bottom of the shallow lake. Even though polyethylene microplastics are less dense than water, the formation of biofilms can change their buoyancy and enable them to settle [66].

Given the history of the George Lake environment, microplastics will likely encounter other pollutants, particularly petroleum-based organic compounds. It is well known that biofilms form on microplastics in aqueous environments, and microplastics have the ability to adsorb a number of other chemicals, especially hydrophobic compounds [67]. A less common scenario is the presence of microplastics in the presence of concentrated amounts of petroleum-based contaminants that result from leaks and spills. A recent study showed the effective adsorption of fuel-based hydrocarbons on microplastics, which can alter the toxicity of the microplastics [68]. This research group collected sediment samples that contained numerous petroleum-based contaminants, including PAHs, open-chain hydrocarbons, and benzene-based compounds. Residents who frequent the George Lake area have reported fuel odors and sheens on the water, which also suggest additional contaminants in the wetland. These additional pollutants complicate the scope of microplastic pollution.

The results from this study suggest that point source microplastic pollution creates greater stress on the environment in the spring season, when pollutant counts are highest and pose a greater threat to wildlife. However, the assumption that the pollutants accumulate during winter requires further sampling to verify this seasonal effect. Sediment analyses showed that the smallest microplastic particles are most vertically mobile through the sediment, especially organic-rich sediments, likely leading to the long-term settling of the pollutants. Ongoing sampling is needed to fully understand the longer-term fate of the microplastics pollution from the manufacturing point source, including the possibility and role of airborne microplastics.

## 4. Conclusions

Microplastics created by a portable toilet manufacturer and released into an adjacent natural wetland were studied and documented from 2021 through July 2023. Polyethylene microplastics contamination was verified by this research group and the state government’s environmental agency. Sampling and analysis were performed at numerous locations in the water, sediment, and air in order to understand the loads, distributions, and fates of the particle contaminants. The data suggest that once released into the open environment, the microplastics move from the company’s property into the marsh and likely remain there until rainfall and other factors facilitate further movement into the lake and sediment. Open lake water samples contained few microplastics, while the load of microplastics in sediment samples was high, especially near the source and along the shoreline. Small microplastics were detected in deeper sediment samples, verifying the vertical movement of these smaller particle pollutants, especially in organic-rich sediments. Preliminary air sampling suggests smaller microplastics are airborne, but more experiments are needed to verify this data. Currently, effective remediation methods do not exist to handle the extensive contamination in the studied wetland and lake. Our proof-of-concept experiments using a novel methodology suggest that it is possible to remove microplastics from water with limited disturbance to natural constituents and before further contamination of the natural area.

## Supplementary Material

Supplementary material

## Data Availability

Data are contained within the article and supplementary materials. Additional supporting materials are available as online documents.
