# Supplementary material for: Distribution and Fate of Polyethylene Microplastics Released by a Portable Toilet Manufacturer into a Freshwater Wetland and Lake
Source: Water (Basel). Author manuscript; Available in PMC 2025 Jan 1. (PMC11361013; doi:10.3390/w16010011)
Supplement: Supplementary material [file NIHMS2000553-supplement-Supplementary_material.pdf]

Supplemental Information for "**Distribution and Fate of Polyethylene Microplastics Released by a Portable Toilet Manufacturer into a Freshwater Wetland and Lake**"

Julie R. Peller [1,*], Gavin Tabor [1], Christina Davis [1], Chris Iceman [1], Ozioma Nwachukwu [2], Kyle Doudrick [2],
Antigone Wilson [1], Alyssa Suprenant [1], David Dabertin [3] and Jon-Paul McCool [4]

[1] Department of Chemistry, Valparaiso University, 1710 Chapel Drive, Valparaiso, IN 46383, USA; gavin.tabor@valpo.edu (G.T.); christina.davis@valpo.edu (C.D.); chris.iceman@valpo.edu (C.I.); antigone.wilson@valpo.edu (A.W.); alyssa.suprenant@valpo.edu (A.S.)
[2] Department of Civil and Environmental Engineering and Earth Sciences, University of Notre Dame, Notre Dame, IN 46556, USA; onwachuk@nd.edu (O.N.); kdoudrick@nd.edu (K.D.)
[3] Dabertin Law Offices, 5246 Hohman Avenue Suite 302, Hammond, IN 46320, USA; dabertin@netnitco.net
[4] Department of Geography and Meteorology, Valparaiso University, 1809 Chapel Drive, Valparaiso, IN 46383, USA; jonpaul.mccool@valpo.edu
* Correspondence: julie.peller@valpo.edu

Table S1. Mass of core sediment samples and microplastic quantities found for sediment samples.

Core #1 – Location 1					
**Depth (cm)**	**Mass Processed (g)**	**Mass MP (mg)**	**Number of Roto/100mL**	**Number of Large Plastic Particles**	**Total MP Count**
0-5	18.8	202.7	~7620	8	7,628
5-10	19.1	170.1	~33	0	33
10-20	30	1.1	~200	1	201
20-30	30	5	250	0	250
**Core #2 – Location 2**					
**Depth (cm)**	**Mass Processed (g)**	**Mass MP (mg)**	**Number of Roto/100mL**	**Number of Large Plastic Particles**	**Total MP Count**
0-5	9.7	182.2	1840	37	1877
5-10	30.0	11.0	492	0	492
10-15	30.0	17.0	480	0	480
15-20	30.0	0.0	15	0	15
20-25	22.2	0.0	6	0	6
**Core #3 – Location 3**					
**Depth (cm)**	**Mass Processed (g)**	**Mass MP (mg)**	**Number of Roto/100mL**	**Number of Large Plastic Particles**	**Total MP Count**
0-5	30	97.1	5660	0	5660
5-10	30	0.0	0	0	0
10-20	30	0.0	4	0	4
20-30	30	0.0	3	0	3
**Core #4 – Location 4**					
**Depth (cm)**	**Mass Processed (g)**	**Mass MP (mg)**	**Number of Roto/100mL**	**Number of Large Plastic Particles**	**Total MP Count**
0-5	3.53	142.9	~2,180	34	~2214
5-10	22.1	0.0	80	0	80
10-20	30	0.0	0	0	0
20-30	30	0.0	0	0	0
**Core #5 – Location 5**					
**Depth (cm)**	**Mass Processed (g)**	**Mass MP (mg)**	**Number of Roto/100mL**	**Number of Large Plastic Particles**	**Total MP Count**
0-5	17.3	0	48	0	48
5-10	17.2	0	3	0	3
10-15	20.9	0	2	0	2
15-20	21.8	0	4	0	4

Table S2. Masses and numbers of microplastics (MP) isolated from 100 mL water samples collected from the George Lake area in April and October 2022 and May 2023. Locations 1 and 2 are the drainage outfall locations, 3 represents the open lake water and locations 4 and 5 are the marsh waters.

Sample location ID (Figure 1)	Mass, MP (g) (April 2022)	MP particles (April 2022)	Mass, MP (g) (October 2022)	MP particles (October 2022)	Mass, MP (g) (May 2023)	MP particles (May 2023)
**1**	**15.530**	**123,680**	**0.0513**	**1643**	**0.431**	**18,594**
**2**	**13.459**	**411,440**	**NA**	**NA**	**0.230**	**3,331**
**3**	**0.745***	**885***	**NA**	**NA**	**0.106**	**726**
**4**	**1.288**	**29,040**	**0.0116***	**4973***	**0.153**	**25,703**
**5**	**0.624**	**8560**	**0.341***	**11,674***	**0.367***	**3232***

***average of two or three samples; NA=not available**

Table S3. Compounds tentatively identified from marsh sediment samples

Extraction/GC-MS	9-dodecyltetrahydroanthracene
Extraction/GC-MS	2,5-dimethyl-phenanthrene
Extraction/GC-MS	9-dodecyltetrahydrophenanthrene
Extraction/GC-MS	7-isopropyl-1,4a-dimethyldecahydronaphthalene
Extraction/GC-MS	BTEX compounds
SPME/GC-MS	3,5-dimethyl-1-hexene
SPME/GC-MS	2-methylbutane
SPME/GC-MS	3-methyl-3-pentanol
SPME/GC-MS	2-propenyloxybenzene

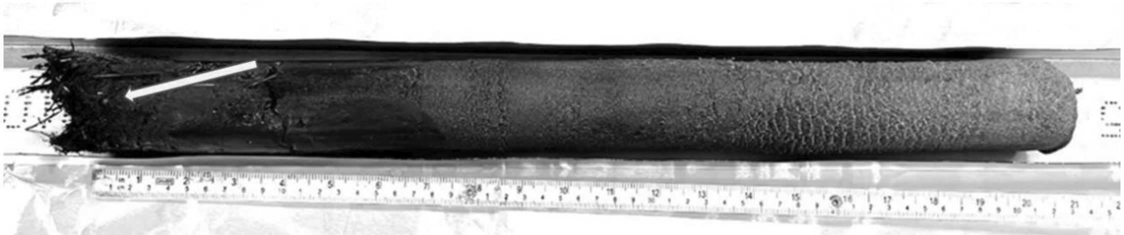

Figure S1. Core 5 sediment core, 52 cm depth. The organic sediment surface on the left contains visible (blue) microplastics. The sandy sediment at lower depths is on the right.

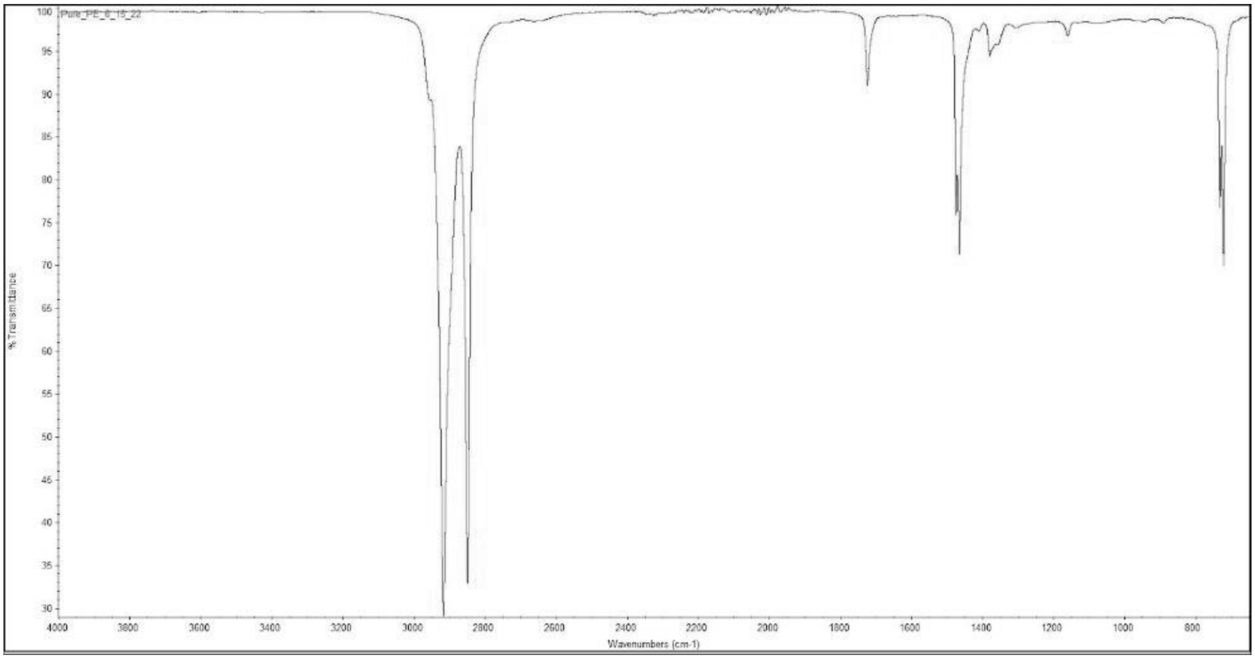

Figure S2. IR spectrum of clean polyethylene recovered from Poly John sample.

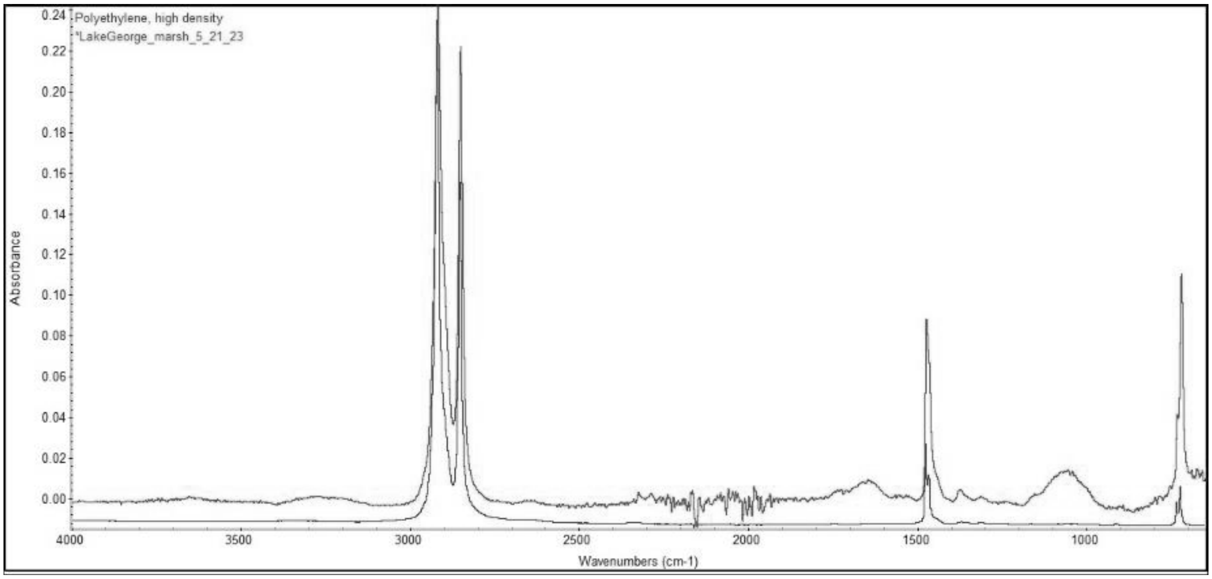

Figure S3. IR spectrum of rotopowder recovered from Poly John sample and library high-density polyethylene spectrum.

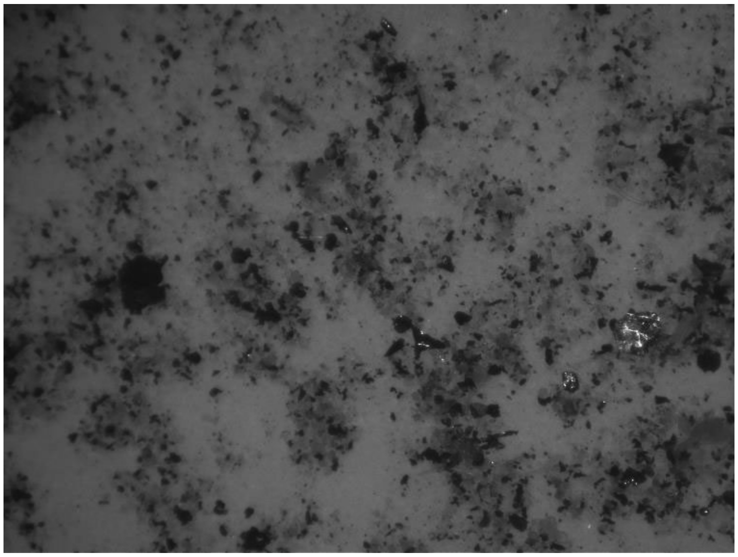

Figure S4. Stereomicroscope image of an oxidized sample from passive atmospheric particle collection near PolyJohn company.

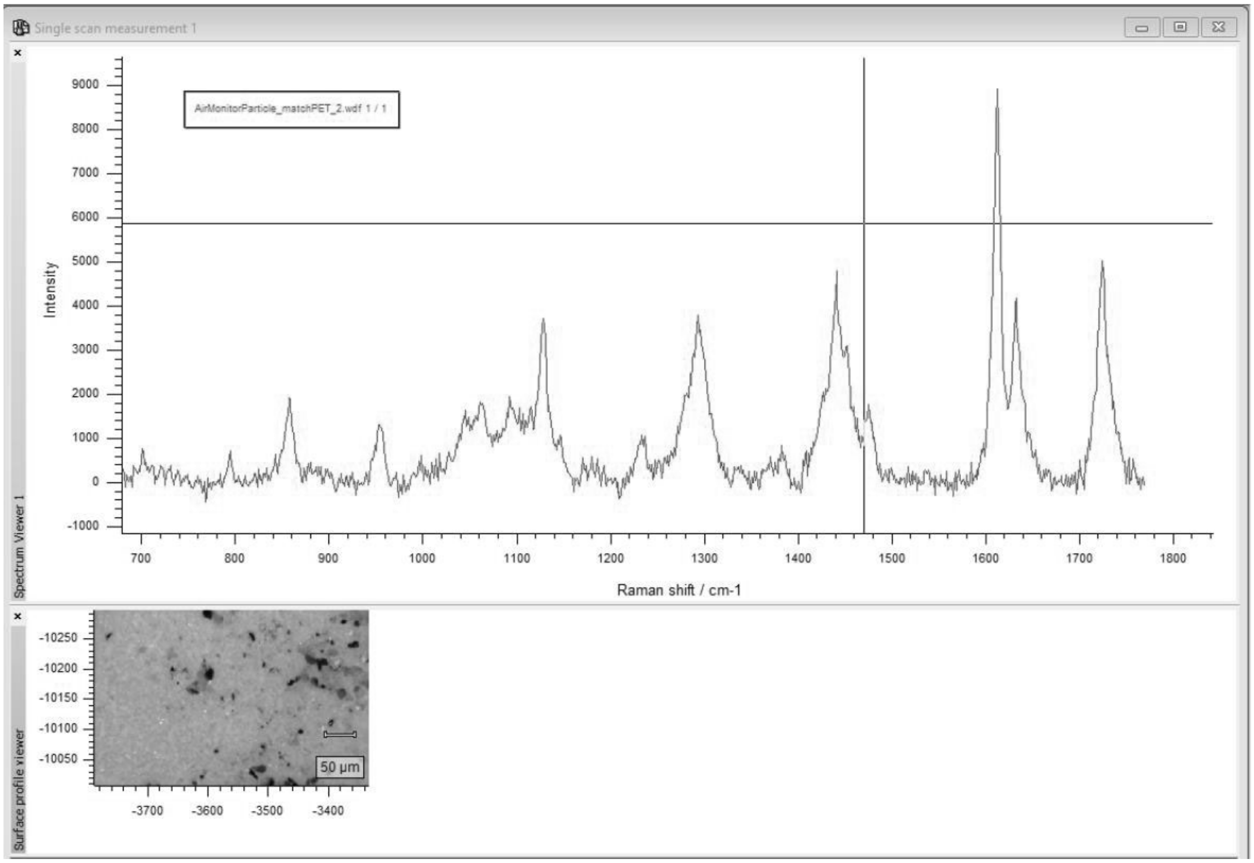

Figure S5. Raman spectrum of air monitor particles on surface of filter. The spectrum matched the library for polyethylene terephthalate, PET.
